# Comorbid mechanisms of psoriasis and mental disorders: an analysis of inflammatory and psychosocial interactions

**DOI:** 10.3389/fpsyt.2026.1807845

**Published:** 2026-07-27

**Authors:** Bi Qin, Huan Pang, Yuhua Huang, Chuntao Chen, Yating Yang, Yangjun Tian, Yan Zhang

**Affiliations:** 1 Department of Dermatology, Institution of Traditional Chinese Medicine of Sichuan Academy of Chinese Medicine Sciences, Chengdu, Sichuan, China; 2 Chengdu University of Traditional Chinese Medicine, Chengdu, Sichuan, China

**Keywords:** comorbidity, mental disorder, pathogenesis, psoriasis, treatment status

## Abstract

**Background:**

Psoriasis is a long-lasting, immune-related inflammatory skin disorder that has been increasingly recognized to be a systemic disorder with significant neuropsychiatric symptoms.

**Objective:**

To critically evaluate the emerging neuroimmune and immune systems that link psoriasis and psychiatric comorbidities, and to assess the implications for treatment.

**Methods:**

A comprehensive literature review was conducted to identify biological, neuroendocrine, and psychosocial pathways linking psoriasis to mental illness. We searched PubMed, Web of Science, and Scopus databases for articles published between January 2000 and December 2025 using keywords including “psoriasis”, “depression”, “anxiety”, “neuroinflammation”, “IL-23/Th17”, and “psychosocial stress”. Studies were included if they addressed human or relevant animal models of psoriasis with psychiatric outcomes, and excluded if they were case reports, editorials, or not published in English. References from retrieved articles were also screened to identify additional relevant studies. This approach ensures reproducibility and minimizes selection bias, consistent with narrative review standards.

**Results:**

The most recent research indicates that psoriasis-related psychological vulnerability is mediated primarily by IL-23/Th17-driven inflammation, dysregulation of the hypothalamic-pituitary-adrenal (HPA) axis, microglial activation, and neuroinflammation. These autoimmune conditions are linked by bidirectional skin-to-brain loops and central-peripheral immune crosstalk, which together lead to cytokine-mediated disruption of neurotransmission and maladaptive stress responses. In addition, stressors in psychosocial contexts and gut and brain interaction have been shown to further exacerbate chronic inflammation, emotional dysregulation, and psychological vulnerability. Immune-targeted biologics and structured psychotherapy may serve as complementary vehicles for the simultaneous treatment of skin inflammation and neurobehavioral outcomes.

**Conclusion:**

This synthesis of mechanisms suggests that neuroimmune circuitry may serve as a unifying construct for understanding psoriasis-related psychiatric illnesses and provides a framework for developing integrated dermatology-psychiatry treatment models. Future work should focus on biomarker-driven, interdisciplinary clinical trials to facilitate the development of tailored treatment approaches.

## 1 Introduction

Psoriasis is a chronic immune-mediated skin disorder characterized by complex interactions among dendritic cells, Th1/Th2/Th17 cells, and B cells, which collectively drive keratinocyte proliferation and sustained cutaneous inflammation. Type 1 immune responses (Th1/IFN-γ) primarily mediate cell-mediated immunity, while Type 2 immune responses (Th2/IL-4, IL-13) contribute to humoral immunity, and Th17/IL-17 pathways play a central role in amplifying local and systemic inflammation.

Psoriasis is driven by the coordinated activity of dendritic cells, Th1, Th2, Th17 cells, and B cells, which collectively promote keratinocyte hyperproliferation and persistent cutaneous inflammation. Type 1 immune responses (Th1/IFN-γ) mediate cell-mediated immunity, Type 2 responses (Th2/IL-4, IL-13) drive humoral immunity, and Th17/IL-17 pathways amplify local and systemic inflammation. Psoriasis also affects all aspects of the patient’s quality of life, including emotional well-being; thus, depression, anxiety, and sleep problems are frequently reported among individuals with psoriasis (1–3).

In recent years, there has been growing evidence describing the biological and clinical links between psoriasis and psychiatric disorders. Early literature discussed epidemiological findings and psychosocial consequences of mobilizing psoriasis; however, emerging data indicate that systemic inflammation, HPA axis dysregulation, and microglial activation within specific brain regions (hippocampus, prefrontal cortex) may contribute to psychiatric comorbidity, with cytokine-mediated neural modulation supported by both clinical biomarker studies and preclinical animal models (4–6). Causal interpretations remain cautious, as human studies are primarily correlational. The role of HPA axis dysregulation and neuroinflammatory processes in psychiatric symptoms in chronic inflammatory disease has generated interest in integrated dermatological and psychiatric care (7).

To date, most literature reviewing the relationship between psoriasis and psychiatric disorders has focused primarily on the co-occurrence of these two conditions and their related symptoms. Recent advances in psychodermatology and neuroimmunology demonstrate that IL-23/Th17-mediated inflammatory pathways, microglial activation, altered neurotransmission, and bidirectional skin–brain immune crosstalk contribute to psychiatric vulnerability in psoriasis patients. Clinical studies report elevated IL-17 and IL-23 serum levels correlating with depression scores, while animal models confirm that Th17 activation can induce neuroinflammatory changes affecting behavior, suggesting a mechanistic link rather than a simple association (8, 9).

Relevant literature was reviewed to identify immune-inflammatory, neuroendocrine, and psychosocial mechanisms linking psoriasis to psychiatric outcomes. “Psychological vulnerability” is defined as an increased risk for mood or anxiety disorders under chronic inflammatory stress, and “stressors in psychosocial contexts” refers to validated life-event or perceived stress measures in human studies (10, 11).

Prior reviews have largely concentrated on the epidemiological associations and symptom burden related to psoriasis. In contrast, this review highlights emerging evidence on neuroimmune circuits, inflammatory cytokine-induced alterations in neurotransmitter systems, and the crosstalk between central and peripheral immune compartments. We also discuss the implications of these findings for the use of immune-targeted biologic therapies and psychotherapy as potential adjunctive treatments for psychiatric symptoms in patients with psoriasis.

This review synthesizes recent mechanistic studies that have identified promising targets for therapeutic intervention. It aims to advance understanding of the psychiatric burden associated with psoriasis and to inform both clinicians and patients in developing personalized, multidisciplinary approaches to care.

## 2 Clinical manifestations and epidemiological patterns of psychiatric comorbidities in psoriasis

### 2.1 Prevalence of psychiatric disorders in psoriasis patients

Psoriasis is a long-lasting skin ailment and is increasingly associated with major depressive disorder (12). It is also linked to generalized anxiety disorder (13) and bipolar disorder (14). Patients with psoriasis frequently experience elevated levels of depressive and anxiety symptoms, which substantially impact quality of life (15). The prevalence of depression among psoriasis patients varies across countries, ranging from 22% in Germany to 30% in China, while anxiety prevalence ranges from 27% in Germany to 34% in China, highlighting regional differences in psychiatric comorbidity (16, 17). Additionally, clinical assessments have identified various factors associated with both depression and anxiety, including disease severity and psychosocial stressors (17). **Table 1** summarizes the reported prevalence of depression and anxiety among psoriasis patients across different countries and study populations.

**Table 1:** Global prevalence of psychiatric disorders in patients with psoriasis.

Country/region	Sample size	Depression prevalence (%)	Anxiety prevalence (%)	Reference
USA	1,200	28	32	(12)
China	1,000	30	34	(13)
Germany	600	22	27	(15)
UK	850	25	29	(16)
Australia	500	26	30	(17)

Patients with psoriasis are at increased risk of sleep disturbances, which are strongly correlated with difficulties in performing daily activities and consequently impair overall quality of life (18, 19). Moreover, suicidal ideation represents an additional concern in this population, with evidence indicating heightened vulnerability among affected individuals (20). Compared with non-affected controls, patients with psoriasis also show higher rates of alcohol use and smoking, behaviors that may reflect maladaptive coping strategies in response to chronic illness-related stress (21, 22). These comorbidities pose barriers to mental health care and treatment adherence, thereby hindering the achievement of clinically meaningful outcomes and underscoring the need for integrated care.

Epidemiological associations are well established, and emerging research suggests that psychological vulnerability in patients with psoriasis arises not from psychosocial stress alone but from shared inflammatory pathways. Systemically elevated cytokine levels (e.g., IL-17, IL-23, TNF-α) in these patients contribute to central nervous system neurotransmitter dysregulation, implying that mood disturbances may be partly mediated by immune-driven changes leading to aberrant neuronal signaling.

### 2.2 Comparative analysis with control populations

Compared with non-psoriatic controls, individuals with psoriasis consistently exhibit greater psychological symptom burden. A prior study found that psoriasis patients had a 2.5-fold increased risk of depression and a 2.0-fold increased risk of anxiety compared with matched controls, with sleep disturbances reported in 35% versus 12% of controls. Severity often parallels inflammatory disease activity (23–25). These differences are associated with substantial impairments in quality of life and psychosocial functioning, underscoring the systemic impact of psoriasis beyond its cutaneous manifestations (26).

Importantly, emerging neuroimaging and biomarker studies reveal altered limbic activation and elevated peripheral inflammatory markers in patients with psoriasis compared with controls, supporting central–peripheral immune crosstalk as a mechanistic substrate for psychiatric comorbidity.

## 3 Contributing factors to psychiatric comorbidities in psoriasis

### 3.1 Disease severity

Psoriasis severity is closely associated with patients’ mental health. Research indicates that individuals with severe psoriasis are more susceptible to psychological problems, including depression, anxiety, and sleep disorders (27). Multivariate analyses have shown that the incidence of depressive and anxiety symptoms is notably higher in patients with severe psoriasis than in those with mild disease (14). Furthermore, biologic therapy in patients with severe psoriasis has been shown not only to ameliorate skin symptoms but also to improve depressive and anxiety symptoms (28). Together, these observations indicate that the severity of psoriasis affects both physical and mental health, reinforcing the importance of early intervention and comprehensive treatment.

### 3.2 Sociodemographic determinants

Sociodemographic factors—including gender, age, income level, and quality of life—substantially influence the prevalence of psychiatric disorders in patients with psoriasis. For instance, studies indicate that female patients tend to experience higher levels of anxiety and depression than their male counterparts, a difference likely attributable to variations in social roles and psychological resilience (29, 30). Age is another crucial factor: younger patients often face greater social pressure and self-image concerns, which increase their risk of mental health problems (31). Moreover, individuals with lower income levels frequently encounter higher rates of mental health disorders, driven by economic strain and limited access to medical resources (32). Declining quality of life is closely correlated with mental health issues; lower quality of life among psoriasis patients is associated with a higher incidence of depression and anxiety, further underscoring the impact of socioeconomic factors on mental well-being (33).

### 3.3 Disease-specific characteristics

Specific features of psoriasis, including itching, lesion location, and psoriatic arthritis, substantially affect patients’ mental health. Itching is one of the most common symptoms in psoriasis patients, and severe itching can exacerbate anxiety and depressive emotions (34, 35). Furthermore, the anatomical location of lesions can influence patients’ self−image; lesions on the face or other exposed areas often lead to more pronounced social anxiety and feelings of inferiority (36).

### 3.4 Integrative biopsychosocial model

Disease severity, sociodemographic stressors, and symptom burden likely converge through shared neuroimmune pathways. Chronic cutaneous inflammation amplifies systemic cytokine load, which, in combination with psychosocial stress, activates the hypothalamic-pituitary-adrenal (HPA) axis and microglial signaling. This integrated biopsychosocial model offers a framework for understanding how external stressors and immune activation synergistically promote psychiatric vulnerability in patients with psoriasis.

## 4 Pathophysiological mechanisms of psychiatric disorders common with psoriasis.

### 4.1 The effects of IL-23/Th17 and neuroinflammation

Psoriasis-related systemic inflammation affects the central nervous system and contributes to psychiatric symptoms through downstream neuroimmune pathways, including microglial activation, disruption of the blood–brain barrier, and neurotransmitter dysregulation. Pro-inflammatory cytokines such as TNF-α, IL-6, and IL-17 increase blood–brain barrier permeability, allowing peripheral signals to access the brain. Subsequently, activated microglia proliferate and release neurotoxic mediators, leading to overstimulation of excitatory neurons and altered synaptic plasticity. These changes ultimately disturb neural network connectivity and are thought to underpin depressive and anxiety symptoms (37–39). Activated microglia alter synapse formation and maintenance and increase excitatory neurotransmitter release; these effects both alter the glutamatergic environment, contributing to neurotoxicity and glutamate imbalance, and provide biological substrates for depression and anxiety due to chronic inflammation (**Table 2**) (39–41).

**Table 2:** Neuroimmune pathways and psychiatric outcomes.

Pathway	Mechanism	Psychiatric outcome	Reference
IL-23/Th17	Cytokine-mediated neuroinflammation	Depression, anxiety	Miller et al., (37)
HPA axis	Cortisol dysregulation	Stress sensitivity, mood disorders	Kiecolt-Glaser et al., (45)
Microglial activation	Synapse remodeling, glutamate imbalance	Cognitive impairment, anxiety	Anisman & Merali, (39)

**Figure 1:**
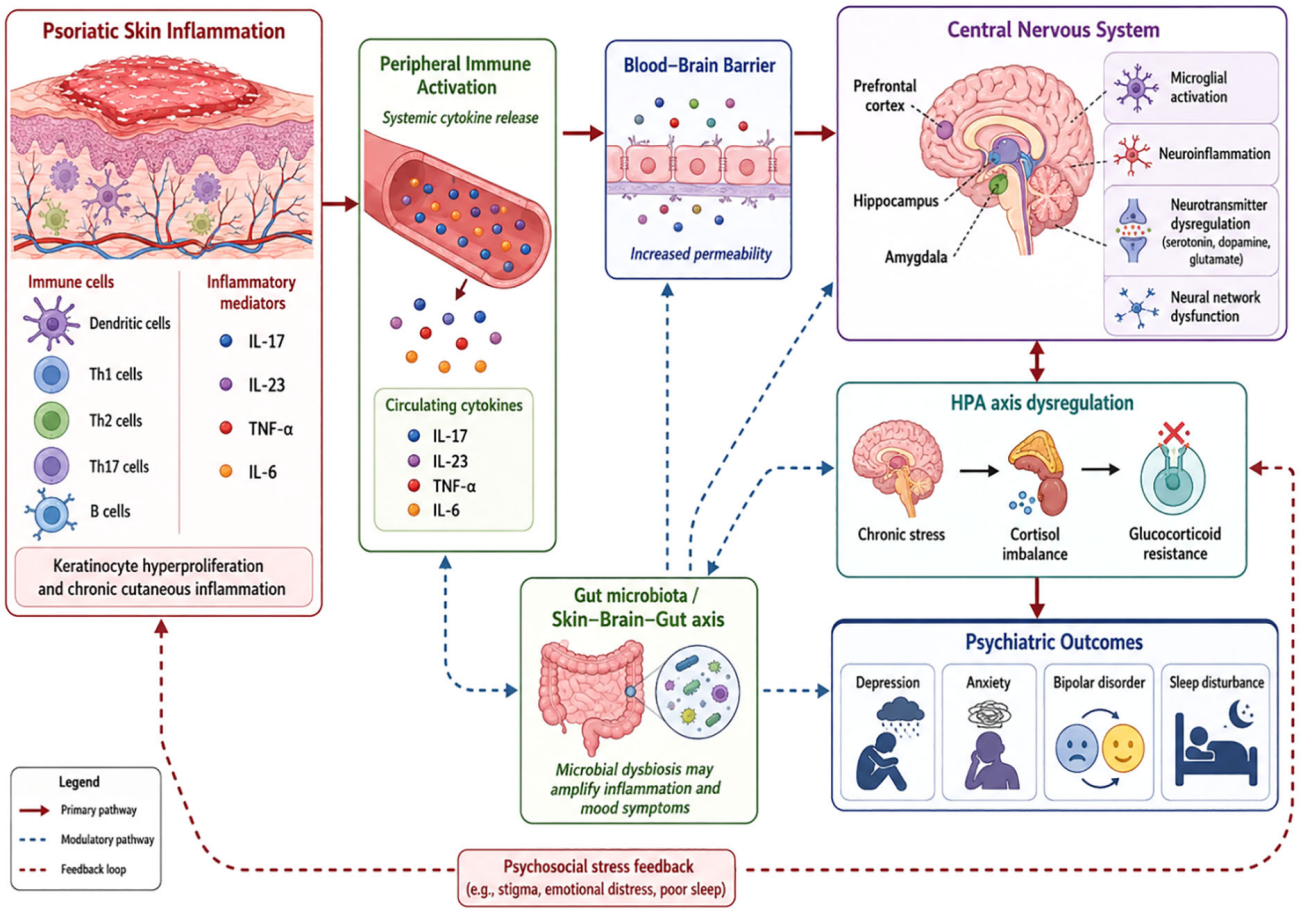
Skin–brain–immune–psychiatric interaction diagram.

Affective symptoms in patients with psoriasis may stem not merely from secondary psychological reactions but directly from immune-mediated neural signaling, implying that neuroimmune circuits constitute a mechanistic link between cutaneous inflammation and psychiatric vulnerability. (**Figure 1**) (41–45).

### 4.2 Cytokine-induced disruption of neurotransmitter systems

Chronic inflammation significantly alters neurotransmission in monoamine systems. These cytokine-mediated changes, stemming from peripheral Type 1/Type 2 immune activation, lead to disruption of serotonin, dopamine, and glutamate signaling, which in turn exacerbates mood disturbances and cognitive impairments. Such neural circuit alterations are linked with observed behavioral phenotypes, including anhedonia, emotional dysregulation, and impaired stress resilience (39, 40, 44, 45). The cytokine-induced alteration of neurotransmitter systems offers a potential explanation for the high prevalence of depressive and anxiety symptoms in psoriasis and other autoimmune disorders.

The notion that disruptions in neurotransmitters resulting from immune-mediated processes could explain the partial resolution of psychiatric symptoms after anti-inflammatory treatments lends empirical support to the view that inflammation is a modifiable determinant of neuro-behavioral outcomes (41–47).

### 4.3 HPA–immune linkage

Chronic systemic inflammation activates the HPA axis in a bi-directional manner. In patients with specific psychiatric disorders, such as major depressive disorder and generalized anxiety disorder, prolonged cytokine signaling induces glucocorticoid resistance and blunts the negative feedback of cortisol, leading to persistent HPA axis overactivation (48, 49). This dysregulation promotes systemic inflammation and neuroendocrine imbalance, amplifying vulnerability to psychiatric symptoms in a subset of patients (46, 47, 50–52). The maladaptive coupling of the HPA axis and immune systems increases the sensitivity of both systems to stress while maintaining an elevated inflammatory state, thereby creating a positive feedback loop that leads to affective instability and anxiety.

Through this mechanism of interaction, psychosocial stressors and immune activation will become biologically linked together, further reinforcing the vulnerability of psychiatric disorders in individuals with an inflammatory disorder (51–54).

### 4.4 Skin–brain–gut axis

Emerging cross-disciplinary evidence suggests that psoriasis-associated psychiatric disorders may involve a tripartite skin–brain–gut axis. The composition of the gut microbiota influences both the immune system and peripheral cytokine production, and also affects central neuroinflammation and mood regulation (54). Consequently, microbial dysbiosis may contribute to both cutaneous inflammation and psychiatric symptoms by modulating peripheral immune status.

Beyond sleep disturbances and chronic stress, peripheral immune signals originating from the gut can fuel neuroinflammation through interconnected feedback loops between the peripheral and central nervous systems (55). This multifaceted skin–brain–gut connection highlights the need for integrated treatment strategies that target immune pathways, modulate the gut microbiota, and enhance psychological resilience to improve quality of life in patients with psoriasis.

## 5 Therapeutic approaches for managing psoriasis and psychiatric comorbidities

### 5.1 Dermatological treatment modalities

#### 5.1.1 Conventional therapies

Conventional psoriasis treatment primarily comprises topical agents, such as corticosteroids and vitamin D3 derivatives, and phototherapy. These methods effectively reduce skin inflammation and scaling, improving patients’ appearance and comfort. Although these treatments focus on skin symptoms, they can also indirectly enhance patients’ self-esteem and social functioning (56, 57). However, given that psoriasis patients often face mental health issues such as depression and anxiety, it is essential to consider the impact of treatment on their mental health to support comprehensive recovery.

#### 5.1.2 Biological therapies

Biological agents not only target autoimmune manifestations but also demonstrate positive influences on mental health. Studies suggest that biologics can reduce inflammation levels, thereby potentially alleviating psychological symptoms such as depression and anxiety (58). Clinical research indicates that ustekinumab and etanercept effectively reduce the severity of psoriasis lesions (59). In a study, those receiving biologics showed a mean reduction of 5.6 points in the PHQ-9 depression score and 4.2 points in the GAD-7 anxiety score over 12 weeks, compared with minimal change in patients not receiving biologics. Overall, the treatment of psoriasis with biologics not only improves skin symptoms but also appears to improve patients’ mental health ratings, suggesting that biologics may enhance mental well-being through immune modulation (58).

### 5.2 Psychiatric interventions and integrated management

Psychotherapy demonstrates substantial benefits for improving mental health. A trial indicates that 8–12 weeks of CBT reduces depression scores by 30–40% and anxiety scores by 25–35% in psoriasis patients, with improvements sustained at 3-month follow-up. Integrated management strategies that combine pharmacotherapy and psychotherapy can offer more comprehensive support, helping patients more effectively manage mental health challenges (60). Furthermore, integrated management strategies that combine pharmacotherapy and psychotherapy can offer more comprehensive support, helping patients more effectively manage mental health challenges. Psychiatric interventions are particularly important in comprehensive treatment and require close collaboration with dermatologists to develop individualized treatment plans that improve patients’ overall physical and mental health.

### 5.3 Comprehensive and multidisciplinary treatment models

The comprehensive treatment model integrates dermatological and psychiatric care to optimize therapeutic outcomes. Personalized strategies that address both physical and psychological needs can improve skin symptoms while alleviating associated depression and anxiety, thereby enhancing overall quality of life. Within this framework, patient education, social support, and family involvement play critical roles in bolstering patient confidence and life satisfaction. Widespread adoption of such integrated approaches is therefore recommended to achieve better outcomes in the management of psoriasis.

### 5.4 Neuro-immune targeted therapeutic implications

Recently, biotherapies targeting Interleukin-17 and Tumor Necrosis Factor Alpha have had the additional effect, in addition to improving symptoms, of reducing both cutaneous inflammation and central neuroinflammatory signaling. This could help explain the reduction in depressive and anxious symptoms observed in patients with psoriasis (58, 59). These therapeutic armamentaria may modulate systemic cytokines and normalize neurotransmitter activity and microglial activation, thereby targeting immune processes to achieve optimal neuropsychiatric outcomes (58).

Psychotherapy, particularly CBT, may dampen inflammatory activity through modulation of the HPA axis, thereby supporting the concept of bidirectional therapeutic effects on psychological and immune regulation (60). However, many psoriasis clinical trials to date lack standardized endpoints for psychiatric disorders or objective neuro-immune markers, which limit mechanistic interpretation of data and hinder the ability to establish tailored treatment priorities for those with psoriasis (58–60).

## 6 Current challenges and future directions

### 6.1 Existing challenges

The diagnosis, treatment, and long-term management of psoriasis with concurrent mental disorders face several challenges. One major issue is the lack of standardized diagnostic criteria for this comorbidity, leading to considerable variation in assessment and treatment across different healthcare facilities. Such inconsistency not only compromises patient outcomes but also results in inefficient use of medical resources.

Another challenge lies in the insufficient attention to individualized treatment planning. Patients with psoriasis often present with complex conditions that include depression or anxiety, requiring holistic consideration of both physiological and psychological states. However, current treatment strategies are typically derived from general population studies and may lack adequate personalization for this specific patient group.

Furthermore, existing research remains limited in elucidating the underlying mechanisms and evaluating treatment efficacy. Although biological links between psoriasis and mental disorders have been identified, the precise pathways remain incompletely understood, hindering the development of targeted therapies. A major limitation of the current literature is the absence of longitudinal neuroimmune profiling and standardized psychiatric outcome measures in psoriasis trials, which precludes causal inference and biomarker-guided therapy (58–60). Addressing these challenges and improving comprehensive management for patients with psoriasis and psychiatric comorbidity therefore represent a key area for continued focus.

### 6.2 Future research directions

Future research should prioritize the identification of emerging biomarkers to better clarify the relationship between mental health and biological factors. Such biomarkers—including inflammatory factors, hormone levels, and gut microbiota composition—could provide a foundation for early diagnosis and personalized treatment by revealing the biological underpinnings of mental health problems.

Multidisciplinary and collaborative clinical research will also play a key role. Integrating insights from psychology, immunology, and neuroscience can deepen our understanding of mental health complexities and offer a scientific basis for developing integrated intervention strategies. To this end, future studies should incorporate cytokine profiling, neuroimaging, and microbiome analyses within prospective cohorts and multidisciplinary clinical trials. This approach will help establish causal neuroimmune pathways and enable personalized intervention strategies, ultimately improving clinical outcomes for patients with psoriasis and psychiatric comorbidities.

## 7 Conclusion

Patients with psoriasis frequently present with psychiatric comorbidities. Accumulating evidence suggests that these conditions are best understood as part of a broader neuro-immune disorder complex, rather than a mere combination of a skin disease and independent psychological problems. A mechanistic framework is emerging in which cutaneous inflammation drives systemic cytokine signaling, neuroendocrine dysregulation, and central neuroinflammatory processes, subsequently altering neurotransmitter levels and affecting mood regulation. Key pathways—including immune cell dysregulation, dysfunction of the HPA axis, psychosocial stress, and the newly recognized skin–brain–gut axis—collectively shape psychiatric risk in individuals with psoriasis.

Effective management of psoriasis requires close collaboration between dermatologists and psychiatrists. Immune-targeted biologic therapies may offer benefits that extend beyond skin clearance by modulating neuroinflammatory pathways, while structured psychotherapeutic interventions can in turn dampen inflammatory components of HPA axis responses to psychosocial stress. This reciprocal interaction creates synergistic opportunities to improve both dermatological outcomes and psychiatric comorbidity.

Future research should focus on biomarker-guided longitudinal studies that integrate cytokine profiling, neuroimaging, and microbiome analysis to establish causal neuroimmune pathways and enable personalized interventions. Translating mechanistic insights into precision-based care for psoriasis patients with psychiatric comorbidity will ultimately depend on interdisciplinary approaches in both science and clinical practice.

## References

[1] Guo J Zhang H Lin W Lu L Su J Chen X . Signaling pathways and targeted therapies for psoriasis. Signal Transduct Target Ther. (2023) 8:437. doi: 10.1038/s41392-023-01655-6. Erratum in: Signal Transduct Target Ther. 2024 ;9(1):25. doi: 10.1038/s41392-023-01733-9. 38253486 PMC10803335

[2] Sahi FM Masood A Danawar NA Mekaiel A Malik BH . Association between psoriasis and depression: a traditional review. Cureus. (2020) 12:e9708. doi: 10.7759/cureus.9708 32944430 PMC7489316

[3] Rendon A Schäkel K . Psoriasis pathogenesis and treatment. Int J Mol Sci. (2019) 20:1475. doi: 10.3390/ijms20061475 30909615 PMC6471628

[4] Wohleb ES Franklin T Iwata M Duman RS . Integrating neuroimmune systems in the neurobiology of depression. Nat Rev Neurosci. (2016) 17:497–511. doi: 10.1038/nrn.2016.69 27277867

[5] Zhang Y Wang J Ye Y Zou Y Chen W Wang Z . Peripheral cytokine levels across psychiatric disorders: a systematic review and network meta-analysis. Prog Neuro-Psychopharmacol Biol Psychiatry. (2023) 125:110740. doi: 10.1016/j.pnpbp.2023.110740 36893912

[6] Sălcudean A Bodo CR Popovici RA Cozma MM Păcurar M Crăciun RE . Neuroinflammation-a crucial factor in the pathophysiology of depression-a comprehensive review. Biomolecules. (2025) 15:502. doi: 10.3390/biom15040502 40305200 PMC12024626

[7] Ferreira BI Abreu JL Reis JP Figueiredo AM . Psoriasis and associated psychiatric disorders: a systematic review on etiopathogenesis and clinical correlation. J Clin Aesthet Dermatol. (2016) 9:36–43. PMC4928455 27386050

[8] Moulton CD Malys M Hopkins CWP Rokakis AS Young AH Powell N . Activation of the interleukin-23/Th17 axis in major depression: a systematic review and meta-analysis. Eur Arch Psychiatry Clin Neurosci. (2025) 275:1653–73. doi: 10.1007/s00406-024-01864-2 39012496 PMC12500783

[9] Beurel E Harrington LE Jope RS . Inflammatory T helper 17 cells promote depression-like behavior in mice. Biol Psychiatry. (2013) 73:622–30. doi: 10.1016/j.biopsych.2012.09.021 23174342 PMC3582833

[10] Fabrazzo M Cipolla S Signoriello S Camerlengo A Calabrese G Giordano GM . A systematic review on shared biological mechanisms of depression and anxiety in comorbidity with psoriasis, atopic dermatitis, and hidradenitis suppurativa. Eur Psychiatry. (2021) 64:e71. doi: 10.1192/j.eurpsy.2021.2249 34819201 PMC8668448

[11] Yang J Zhang S Wu Q Chen P Dai Y Long J . T cell-mediated skin-brain axis: bridging the gap between psoriasis and psychiatric comorbidities. J Autoimmun. (2024) 144:103176. doi: 10.1016/j.jaut.2024.103176 38364575

[12] Parisi R Webb RT Kleyn CE Carr MJ Kapur N Griffiths CEM . Psychiatric morbidity and suicidal behaviour in psoriasis: a primary care cohort study. Br J Dermatol. (2019) 180:108–15. doi: 10.1111/bjd.17004 30007069

[13] Chu M Shen S Zhu Z Li Z Bai Y Ma J . Association of psoriasis with depression, anxiety, and suicidality: a bidirectional two-sample Mendelian randomization study. J Dermatol. (2023) 50:1629–34. doi: 10.1111/1346-8138.16941 37697936

[14] Kurd SK Troxel AB Crits-Christoph P Gelfand JM . The risk of depression, anxiety, and suicidality in patients with psoriasis: a population-based cohort study. Arch Dermatol. (2010) 146:891–5. doi: 10.1001/archdermatol.2010.186 20713823 PMC2928071

[15] McDonough E Ayearst R Eder L Chandran V Rosen CF Thavaneswaran A . Depression and anxiety in psoriatic disease: prevalence and associated factors. J Rheumatol. (2014) 41:887–96. doi: 10.3899/jrheum.130797 24692521

[16] Fleming P Bai JW Pratt M Sibbald C Lynde C Gulliver WP . The prevalence of anxiety in patients with psoriasis: a systematic review of observational studies and clinical trials. J Eur Acad Dermatol Venereol. (2017) 31:798–807. doi: 10.1111/jdv.13891 27620704

[17] Pollo CF Miot HA Matos TDS de Souza JM Jorge MFS Miot LDB . Prevalence and factors associated with depression and anxiety in patients with psoriasis. J Clin Nurs. (2021) 30:572–80. doi: 10.1111/jocn.15577 33258200

[18] Smith P Jin JQ Spencer RK Elhage KG Johnson CE Haran K . Psoriasis and sleep disturbance: a US population-based study using the NHANES database. Dermatol Ther (Heidelb). (2024) 14:2277–83. doi: 10.1007/s13555-024-01211-2 38940897 PMC11333390

[19] Nowowiejska J Baran A Flisiak I . Mutual relationship between sleep disorders, quality of life and psychosocial aspects in patients with psoriasis. Front Psychiatry. (2021) 12:674460. doi: 10.3389/fpsyt.2021.674460 34295272 PMC8290261

[20] Liang SE Cohen JM Ho RS . Psoriasis and suicidality: a review of the literature. Dermatol Ther. (2019) 32:e12771. doi: 10.1111/dth.12771 30315629

[21] Temiz SA Özer İ Ataseven A Dursun R Uyar M . The effect of smoking on the psoriasis: is it related to nail involvement? Dermatol Ther. (2020) 33:e13960. doi: 10.1111/dth.13960 32621631

[22] Adamzik K McAleer MA Kirby B . Alcohol and psoriasis: sobering thoughts. Clin Exp Dermatol. (2013) 38:819–22. doi: 10.1111/ced.12013 24252076

[23] Grattan RE Linscott RJ . Components of schizophrenia liability affect the growth of psychological stress sensitivity following major life events. Schizophr Res. (2019) 212:134–9. doi: 10.1016/j.schres.2019.07.056 31387827

[24] Pan Z Zhang D . Relationship between stressful life events and sleep quality: the mediating and moderating role of psychological suzhi. Sleep Med. (2022) 96:28–34. doi: 10.1016/j.sleep.2022.04.026 35576831

[25] Sharma VK Singh TG . Chronic stress and diabetes mellitus: interwoven pathologies. Curr Diabetes Rev. (2020) 16:546–56. doi: 10.2174/1573399815666191111152248 31713487

[26] He C Morris N McNiel D Binder R . The evolving standard of care for autoimmune neuropsychiatric illness. J Am Acad Psychiatry Law. (2024) 52:225–34. 10.29158/JAAPL.240033-24 38824424

[27] Tribó MJ Turroja M Castaño-Vinyals G Bulbena A Ros E García-Martínez P . Patients with moderate to severe psoriasis associate with higher risk of depression and anxiety symptoms: results of a multivariate study of 300 Spanish individuals with psoriasis. Acta Derm Venereol. (2019) 99:417–22. doi: 10.2340/00015555-3114 30628634

[28] Uğurer E Altunay İK Özkur E Baltan E . The effects of methotrexate and biologics on the symptoms of depression and anxiety in patients with psoriasis. Indian J Dermatol. (2023) 68:237–44. doi: 10.4103/ijd.ijd_241_22 37529469 PMC10389124

[29] Golpour M Hosseini SH Khademloo M Ghasemi M Ebadi A Koohkan F . Depression and anxiety disorders among patients with psoriasis: a hospital-based case-control study. Dermatol Res Pract. (2012) 2012:381905. doi: 10.1155/2012/381905 22844272 PMC3403219

[30] Schmid-Ott G . Causality for depression and psoriasis is a sex-specific two-way process. Br J Dermatol. (2015) 173:891. doi: 10.1111/bjd.14096 26511826

[31] Michail M Mughal F Robinson J . Suicide prevention in young people: optimising primary care. Br J Gen Pract. (2020) 70:104–5. doi: 10.3399/bjgp20X708329 32107216 PMC7038832

[32] Patel V Kleinman A . Poverty and common mental disorders in developing countries. Bull World Health Organ. (2003) 81:609–15. doi: 10.1037/e538812013-019 14576893 PMC2572527

[33] Kim GE Seidler E Kimball AB . A measure of chronic quality of life predicts socioeconomic and medical outcomes in psoriasis patients. J Eur Acad Dermatol Venereol. (2015) 29:249–54. doi: 10.1111/jdv.12503 24684416

[34] Reich A Mędrek K Szepietowski JC . Interplay of itch and psyche in psoriasis: an update. Acta Derm Venereol. (2016) 96:55–7. doi: 10.2340/00015555-2374 27282194

[35] Ograczyk A Miniszewska J Kępska A Zalewska-Janowska A . Itch, disease coping strategies and quality of life in psoriasis patients. Postepy Dermatol Alergol. (2014) 31:299–304. doi: 10.5114/pdia.2014.40927 25395926 PMC4221347

[36] Łakuta P Marcinkiewicz K Bergler-Czop B Brzezińska-Wcisło L Słomian A . Associations between site of skin lesions and depression, social anxiety, body-related emotions and feelings of stigmatization in psoriasis patients. Postepy Dermatol Alergol. (2018) 35:60–6. doi: 10.5114/pdia.2016.62287 29599673 PMC5872236

[37] Miller AH Haroon E Raison CL Felger JC . Cytokine targets in the brain: impact on neurotransmitters and neurocircuits. Depress Anxiety. (2013) 30:297–306. doi: 10.1002/da.22084 23468190 PMC4141874

[38] Bajetto A Bonavia R Barbero S Schettini G . Characterization of chemokines and their receptors in the central nervous system: physiopathological implications. J Neurochem. (2002) 82:1311–29. doi: 10.1046/j.1471-4159.2002.01091.x 12354279

[39] Anisman H Merali Z . Cytokines, stress and depressive illness: brain-immune interactions. Ann Med. (2003) 35:2–11. doi: 10.1080/07853890310004075 12693607

[40] Kalkman HO . Novel treatment targets based on insights in the etiology of depression: role of IL-6 trans-signaling and stress-induced elevation of glutamate and ATP. Pharm (Basel). (2019) 12:113. doi: 10.3390/ph12030113 31362361 PMC6789839

[41] Chang HH Chen PS . Inflammatory biomarkers for mood disorders - a brief narrative review. Curr Pharm Des. (2020) 26:236–43. doi: 10.2174/1381612826666200115100726 31939727

[42] Kojima M Kojima T Suzuki S Oguchi T Oba M Tsuchiya H . Depression, inflammation, and pain in patients with rheumatoid arthritis. Arthritis Rheum. (2009) 61:1018–24. doi: 10.1002/art.24647 19644894

[43] Spiegel DR Holtz L Chopra K . A case of mania in a patient with systemic lupus erythematosus: can its inflammatory pathogenesis be applied to primary mood disorders? Psychiatry (Edgmont). (2010) 7:31–6. PMC2877619 20508806

[44] Ironside M Admon R Maddox SA Mehta M Douglas S Olson DP . Inflammation and depressive phenotypes: evidence from medical records from over 12 000 patients and brain morphology. Psychol Med. (2020) 50:2790–8. doi: 10.1017/S0033291719002940 31615590 PMC7160032

[45] Kiecolt-Glaser JK Derry HM Fagundes CP . Inflammation: depression fans the flames and feasts on the heat. Am J Psychiatry. (2015) 172:1075–91. doi: 10.1176/appi.ajp.2015.15020152 26357876 PMC6511978

[46] Gama JFG Cardoso LMDF Bisaggio RDC Lagrota-Candido J Henriques-Pons A Alves LA . Immunological tolerance in liver transplant recipients: putative involvement of neuroendocrine-immune interactions. Cells. (2022) 11:2327. doi: 10.3390/cells11152327 35954171 PMC9367574

[47] Vela-Patiño S Salazar MI Remba-Shapiro I Peña-Martínez E Silva-Roman G Andoneui-Elguera S . Neuroendocrine-immune interface: interactions of two complex systems in health and disease. Arch Med Res. (2022) 53:240–51. doi: 10.1016/j.arcmed.2022.01.003 35153080

[48] Heim C Mletzko T Purselle D Musselman DL Nemeroff CB . The dexamethasone/corticotropin-releasing factor test in men with major depression: role of childhood trauma. Biol Psychiatry. (2008) 63:398–405. doi: 10.1016/j.biopsych.2007.07.002 17825799

[49] Hacimusalar Y Esel E . Evaluation of hypothalamo-pituitary-adrenal axis activity by using dexamethasone suppression test in patients with panic disorder and generalized anxiety disorder. Dusunen Adam J Psychiatry Neurol Sci. (2017) 30:15–24. doi: 10.5350/dajpn2017300102

[50] Hinchcliffe JK Mendl M Robinson ESJ . Investigating hormone-induced changes in affective state using the affective bias test in male and female rats. Psychoneuroendocrinology. (2020) 115:104647. doi: 10.1016/j.psyneuen.2020.104647 32179367 PMC7193894

[51] Breznoscakova D Pallayova M . Case report: Uncovering hidden glucose patterns in medicated versus unmedicated bipolar disorder and comorbid type 1 diabetes mellitus. Front Endocrinol (Lausanne). (2024) 15:1354749. doi: 10.3389/fendo.2024.1354749 38419952 PMC10899695

[52] Papa V Li Pomi F Borgia F Genovese S Pioggia G Gangemi S . Mens sana in cute sana"-a state of the art of mutual etiopathogenetic influence and relevant pathophysiological pathways between skin and mental disorders: An integrated approach to contemporary psychopathological scenarios. Cells. (2023) 12:1828. doi: 10.3390/cells12141828 37508493 PMC10377895

[53] Belinati Loureiro V Ratzke R Nogueira Dutra JC Mesadri Gewehr D Cantilino A Pinto da Costa M . Psychotherapy training in Brazil: Experiences of psychiatric trainees and early career psychiatrists. Med (Baltimore). (2023) 102:e35388. doi: 10.1097/MD.0000000000035388 38115245 PMC10727625

[54] Hitch C Leightley D Murphy D Trompeter N Dymond S . Acceptance and commitment therapy for co-occurring gambling disorder and posttraumatic stress disorder in veterans: A narrative review. Eur J Psychotraumatol. (2023) 14:2178203. doi: 10.1080/20008066.2023.2178203 37052089 PMC9970237

[55] Lin LP Tan MTT . Biosensors for the detection of lung cancer biomarkers: A review on biomarkers, transducing techniques and recent graphene-based implementations. Biosens Bioelectron. (2023) 237:115492. doi: 10.1016/j.bios.2023.115492 37421797

[56] Soleymani T Hung T Soung J . The role of vitamin D in psoriasis: A review. Int J Dermatol. (2015) 54:383–92. doi: 10.1111/ijd.12790 25601579

[57] Paul C Ortonne JP . Topical treatment and phototherapy in psoriasis: Systematic review and expert opinion of a panel of dermatologists. J Eur Acad Dermatol Venereol. (2012) 26:iii–iv. doi: 10.1111/j.1468-3083.2012.04517.x 22512683

[58] Timis TL Beni L Mocan T Florian IA Orasan RI . Biologic therapies decrease disease severity and improve depression and anxiety symptoms in psoriasis patients. Life (Basel). (2023) 13:1219. doi: 10.3390/life13051219 37240864 PMC10220716

[59] Aslam N Saleem H Murtazaliev S Quazi SJ Khan S . FDA approved biologics: Can etanercept and ustekinumab be considered a first-line systemic therapy for pediatric/adolescents in moderate to severe psoriasis? A systematic review. Cureus. (2020) 12:e9812. doi: 10.7759/cureus.9812 32953323 PMC7494414

[60] Gallagher MW Phillips CA D'Souza J Richardson A Long LJ Boswell JF . Trajectories of change in well-being during cognitive behavioral therapies for anxiety disorders: Quantifying the impact and covariation with improvements in anxiety. Psychother (Chic). (2020) 57:379–90. doi: 10.1037/pst0000283 32027157 PMC7416465

